# Cardiovascular Magnetic Resonance in Survivors of Critical Illness: Cardiac Abnormalities Are Associated With Acute Kidney Injury

**DOI:** 10.1161/JAHA.123.029492

**Published:** 2023-04-29

**Authors:** Alexander Isaak, Isabel Pomareda, Narine Mesropyan, Dmitrij Kravchenko, Christoph Endler, Leon Bischoff, Claus C. Pieper, Daniel Kuetting, Ulrike Attenberger, Sebastian Zimmer, Christian Putensen, Jens‐Christian Schewe, Stefan Kreyer, Julian A. Luetkens

**Affiliations:** ^1^ Department of Diagnostic and Interventional Radiology University Hospital Bonn Bonn Germany; ^2^ Quantitative Imaging Lab Bonn (QILaB) University Hospital Bonn Bonn Germany; ^3^ Clinic for Internal Medicine II, Heart Center Bonn University Hospital Bonn Bonn Germany; ^4^ Department of Anesthesiology and Intensive Care Medicine University Hospital Bonn Bonn Germany; ^5^ Department of Anesthesiology, Intensive Care Medicine and Pain Therapy University Medical Centre Rostock Rostock Germany

**Keywords:** acute kidney injury, cardiorenal syndrome, critical illness, intensive care unit, magnetic resonance imaging, post–intensive care syndrome, Fibrosis, Nephrology and Kidney, Cardiorenal Syndrome, Cardiomyopathy, Magnetic Resonance Imaging (MRI)

## Abstract

**Background:**

The objective of this study was to investigate cardiac abnormalities in intensive care unit (ICU) survivors of critical illness and to determine whether temporary acute kidney injury (AKI) is associated with more pronounced findings on cardiovascular magnetic resonance.

**Methods and Results:**

There were 2175 patients treated in the ICU (from 2015 until 2021) due to critical illness who were screened for study eligibility. Post‐ICU patients without known cardiac disease were prospectively recruited from March 2021 to May 2022. Participants underwent cardiovascular magnetic resonance including assessment of cardiac function, myocardial edema, late gadolinium enhancement, and mapping including extracellular volume fraction. Student *t* test, Mann‐Whitney *U* test, and χ^2^ tests were used. There were 48 ICU survivors (46±15 years of age, 28 men, 29 with AKI and continuous kidney replacement therapy, and 19 without AKI) and 20 healthy controls who were included. ICU survivors had elevated markers of myocardial fibrosis (T1: 995±31 ms versus 957±21 ms, *P*<0.001; extracellular volume fraction: 24.9±2.5% versus 22.8±1.2%, *P*<0.001; late gadolinium enhancement: 1% [0%–3%] versus 0% [0%–0%], *P*<0.001), more frequent focal late gadolinium enhancement lesions (21% versus 0%, *P*=0.03), and an impaired left ventricular function (eg, ejection fraction: 57±6% versus 60±5%, *P*=0.03; systolic longitudinal strain: 20.3±3.7% versus 23.1±3.5%, *P*=0.004) compared with healthy controls. ICU survivors with AKI had higher myocardial T1 (1002±33 ms versus 983±21 ms; *P*=0.046) and extracellular volume fraction values (25.6±2.6% versus 23.9±1.9%; *P*=0.02) compared with participants without AKI.

**Conclusions:**

ICU survivors of critical illness without previously diagnosed cardiac disease had distinct abnormalities on cardiovascular magnetic resonance including signs of myocardial fibrosis and systolic dysfunction. Findings were more abnormal in participants who experienced AKI with necessity of continuous kidney replacement therapy during their ICU stay.

**Registration:**

URL: https://www.clinicaltrials.gov; Unique identifier: NCT 05034588.

**Nonstandard Abbreviations and Acronyms**
AKI: acute kidney injury
CKRT: continuous kidney replacement therapy
CRS: cardiorenal syndrome
ECV: extracellular volume fraction
GLS: global longitudinal strain
LGE: late gadolinium enhancement

**Clinical Perspective**

**What Is New?**

- Survivors of critical illness without prior known cardiac disease showed mildly impaired parameter of left ventricular function and elevated markers of myocardial fibrosis in the absence of myocardial edema (frequency of late gadolinium enhancement lesions: 21% versus 0%, *P*=0.03; T1 relaxation time: 995±31 ms versus 957±21 ms, *P*<0.001; extracellular volume fraction: 24.9±2.5% versus 22.8±1.2%, *P*<0.001) compared with healthy controls.
- Myocardial markers were more abnormal in participants after transient acute kidney injury (T1 relaxation time: 1002±33 ms versus 983±21 ms, *P*=0.046; extracellular volume fraction: 25.6±2.6% versus 23.9±1.9%, *P*=0.02).

**What Are the Clinical Implications?**

- Unrecognized fibrotic and functional myocardial abnormalities in intensive care unit survivors may be associated with physical impairment in post–intensive care syndrome.
- These findings further support current theories of a contribution of acute kidney injury to the development of functional and structural cardiac abnormalities (cardiorenal syndrome type 3).
- In the present era, it is important to highlight that such cardiac abnormalities may generally occur in intensive care unit survivors, regardless of COVID‐19 disease.

Critical illness syndromes are characterized by different acutely life‐threatening clinical conditions that usually require intensive care unit (ICU) treatment and, if survived, can lead to physical, cognitive, and psychological impairment.^1^ Several mechanisms promoting cardiovascular disease in response to critical illness have been described.^2^ Immunometabolic changes, sustained inflammatory cascades, and activation of neurohormonal signaling pathways appear to contribute to fibrotic cardiac remodeling, atherosclerosis, and cardiac dysfunction.^3^ Specifically, increased exposure to catecholamines, oxidative stress, and altered mitochondrial function were found to be involved in these changes.^3^, ^4^ In a previous study, biomarkers of cardiac failure were associated with reduced long‐term survival in patients after ICU treatment.^5^ Further studies indicate an increased risk of cardiovascular events in post‐ICU patients with severe sepsis.^6^, ^7^

Severe sepsis is often accompanied by acute kidney injury (AKI), a condition that is one of the most common complications of critical illness.^8^ AKI is a predictor of short‐ and long‐term adverse cardiovascular events^9^ and increases the risk of cardiovascular mortality by 86%.^10^ Reciprocal effects between cardiac and kidney disease are referred to as cardiorenal syndrome (CRS), with type 3 CRS describing cardiac disease as a result of AKI.^11^ In AKI, hemodynamic and metabolic alterations, and activation of the renin‐angiotensin‐aldosterone system and inflammatory pathways are associated with direct cardiodepressant effects.^9^ The most commonly described cardiac sequelae after AKI are congestive heart failure and acute myocardial infarction.^10^, ^12^ Severe cases of AKI, which often require continuous kidney replacement therapy (CKRT), appear to have a particularly negative impact on this outcome.^13^, ^14^ However, the extent and manner in which critical illness and AKI contribute to myocardial injury requires further investigation. Previous cardiovascular magnetic resonance (CMR) studies have already detected myocardial tissue alterations in patients with chronic kidney disease (type 4 CRS).^15^, ^16^

In this cross‐sectional study, CMR was performed in ICU survivors of critical illness without previously known cardiac disease to investigate the extent of subclinical myocardial abnormalities such as fibrosis, inflammation, or ventricular dysfunction. Based on the latest evidence of type 3 CRS,^9^ we hypothesized that AKI during ICU treatment would lead to more pronounced myocardial abnormalities.

## Methods

The data that support the findings of this study are available from the corresponding author upon reasonable request. This cross‐sectional study was approved by the institutional ethics committee (approval ID: 519/20). All participants gave written informed consent before CMR. The study is registered at clinicaltrials.gov (identifier: NCT 05034588).

### Study Participants

Survivors of critical illness with past treatment in the ICU were screened for study eligibility. Patients with ICU treatment of >3 days at the Department of Anesthesiology and Intensive Care Medicine (University Hospital Bonn) between January 2015 and December 2021 due to critical illness (defined as an acute life‐threatening illness requiring intensive care [eg, trauma, hemorrhage, stroke, infection, respiratory failure])^2^ were identified and evaluated for potential study participation. Only participants without previously diagnosed cardiac disease or systemic disease with potential cardiac involvement (eg, sarcoidosis, amyloidosis, autoimmune/inflammatory diseases) before, during, and after ICU treatment were included. Further exclusion criteria were contraindications for contrast‐enhanced CMR. Participants prospectively underwent CMR from March 2021 to May 2022.

Post‐ICU participants were subdivided into (1) participants who had recovered from AKI with necessity of CKRT during critical illness (AKI group), and (2) participants without acute or chronic kidney injury during critical illness (non‐AKI group). Only participants with history of AKI including temporary CKRT (stage 3 according to Kidney Disease: Improving Global Outcomes) with subsequent recovery of kidney function after ICU treatment were included. Convalescence was defined according to the Kidney Disease: Improving Global Outcomes guidelines as glomerular filtration rate ≥60 mL/min per 1.73 m^2^ in the absence of CKRT.^17^ To exclude patients with chronic kidney disease and due to the institutional ethics regulations, only participants with a glomerular filtration rate ≥45 mL/min per 1.73 m^2^ at the time of MRI were included. Intensive care scores (Simplified Acute Physiology Score II [excluding Glasgow Coma Scale calculation] and Therapeutic Intervention Scoring System‐10) were derived from medical records. The control group consisted of healthy subjects without previous ICU stay and no cardiac disease history who underwent CMR for study control reasons. Controls had normal CMR results without structural abnormalities.

### CMR Protocol

All CMR examinations were performed using a clinical 1.5T whole‐body system (1.5 Ingenia; Philips Medical Systems, Best, the Netherlands). ECG‐gated steady state free‐precession cine sequences, T2‐weighted short‐τ inversion‐recovery sequences, and late gadolinium enhancement (LGE) based on segmented inversion recovery gradient echo sequences were acquired in standard orientations. Myocardial T1 and T2 mapping was obtained in apical, midventricular, and basal end‐diastolic short‐axis view using the modified look‐locker inversion recovery scheme and the gradient and spin‐echo sequence sequence.^18^ For contrast enhancement, a bolus of 0.2 mmol/kg body weight of gadoterate meglumine (Clariscan; GE Healthcare) was administered.

### Image Analysis

Two experienced cardiovascular radiologists (J.A.L., A.I.) performed image analysis in consensus agreement blinded to the clinical data using appropriate software (IntelliSpace Portal Version 12; Philips Medical System). Functional analysis including the assessment of ventricular volumes, function, mass, and feature‐tracking strain (systolic global longitudinal [GLS], global circumferential strain, and global radial strain) were analyzed as previously described.^18^, ^19^ LGE was assessed visually (presence of enhancement) and semiquantitatively (full width at half maximum method).^19^ Myocardial edema was evaluated visually (presence of regional hyperintensity on T2‐weighted images) and quantitatively using T2 mapping.^18^, ^20^ Myocardial T1 relaxation times and hematocrit corrected extracellular volume fraction (ECV) were calculated, as previously described.^18^, ^20^ T1, T2, and ECV values were measured using a global approach.

### Statistical Analysis

Statistical analysis was performed using SPSS Statistics (version 26; IBM, Armonk, NY) and Prism (version 8.4.3; GraphPad Software). Participant characteristics are reported as mean±standard deviation, median and interquartile range (IQR), or as percentages and absolute frequencies. Normal distribution was assessed visually using normal distribution plots and supplemented by the Shapiro‐Wilk test. Continuous variables were compared using the independent 2‐sample Student *t* test (normally distributed variables) or the Mann‐Whitney *U* test (not normally distributed variables). Independence between dichotomous variables was tested using the χ^2^ test (when cell count >5) and Fisher exact test (when cell count ≤5). One‐way analysis of variance with subsequent Tukey multiple comparison tests was performed to compare CMR characteristics between the ICU subgroups and healthy controls. Continuous nonparametric variables between the 2 ICU subgroups were compared using the Kruskal‐Wallis test. Correlations between continuous variables were tested using Pearson correlation coefficients. The level of statistical significance was set at *P*<0.05.

## Results

### General Characteristics of ICU Survivors

**Figure 1:**
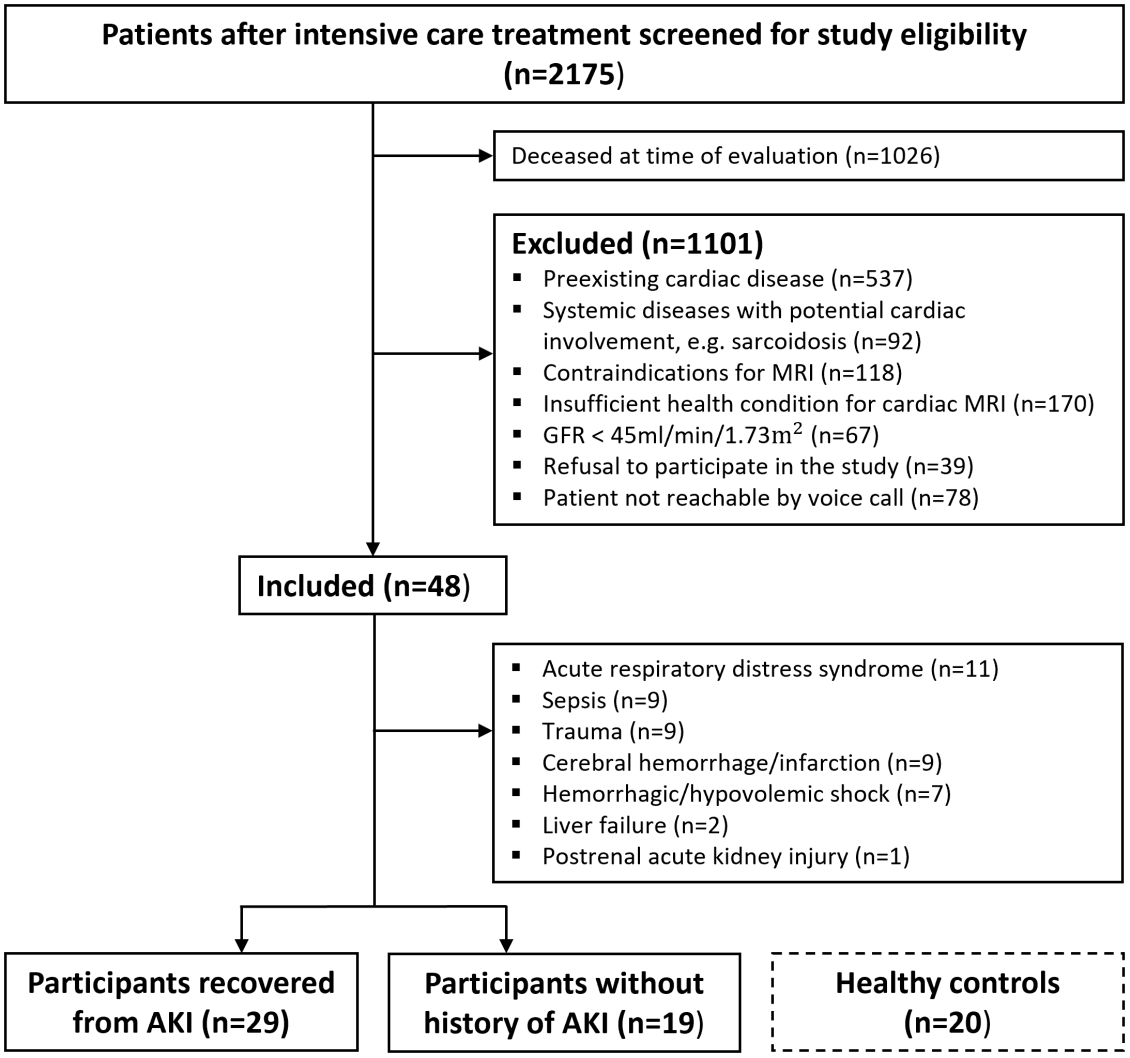
Study flowchart. AKI indicates acute kidney injury; GFR, glomerular filtration rate; and MRI, magnetic resonance imaging.

A total of 2175 patients with past ICU treatment due to critical illness was retrospectively screened for study eligibility (Figure 1). From March 2021 to May 2022, 68 participants prospectively underwent CMR: 48 ICU survivors of critical illness (mean age±standard deviation, 46±15 years; 42% women) and 20 healthy control participants (mean age±standard deviation, 48±14 years; 45% women). The main reasons for ICU admission were acute respiratory distress syndrome (11/48, 23%), sepsis (9/48, 19%), trauma (9/48, 19%), cerebral hemorrhage/infarction (9/48, 19%), and hemorrhagic/hypovolemic shock (4/48, 8%). Thirteen of 48 participants (27%) had ICU treatment during the COVID‐19 pandemic (since March 2020), and 8 of 48 participants (16%) had COVID‐19 during ICU hospitalization. The median length of ICU stay was 35 days (IQR, 22–58 days). The median interval between hospital discharge and CMR scan was 27 months (IQR, 13–50 months).

The AKI group consisted of 29 of 48 (60%) participants, and the non‐AKI group consisted of 19 of 48 (40%) participants. The AKI group received temporary CKRT over a median duration of 12 days (IQR, 6–36 days). Over the clinical course, return of normal kidney function was observed in all AKI participants, as defined by the Kidney Disease: Improving Global Outcomes guidelines (median of the last measured glomerular filtration rate before CMR, 96 mL/min per 1.73 m^2^; IQR, 72–117 mL/min per 1.73 m^2^). AKI and non‐AKI participants did not differ in terms of length of ICU stay, duration of mechanical ventilation, or intensive care scoring; however, the AKI group had higher absolute scores in all these categories (Table 1). Twelve of 48 participants (25%) received extracorporeal membrane oxygenation treatment. In participants with available troponin values (36/48, 75%), troponin levels were elevated in 26 of 36 ICU survivors (72%), without a significant difference between the AKI and non‐AKI group (17/22 [77%] versus 9/14 [64%]; *P*=0.40). Troponin peak values showed no significant difference between the AKI and non‐AKI group (Table 1). There was no significant difference found in cardiovascular risk factors between ICU survivors with and without AKI (Table 1).

**Table 1:** Clinical Characteristics of ICU Survivors of Critical Illness, Consisting of Participants Who Recovered From Acute Kidney Injury and Participants Without History of AKI

Parameter	Total ICU survivors, n=48	ICU survivors AKI+, n=29	ICU survivors AKI−, n=19	*P* value, AKI+ vs AKI−
General parameters				
Age, y	46±15	45±15	48±14	0.87
Sex, women	20 (42%)	13 (45%)	7 (37%)	0.58
Body mass index, kg/m^2^	28±4	27±4	28±5	0.72
Heart rate, bpm	67±10	68±11	67±11	0.96
ICU parameters				
ICU length of stay, d	35 (22–58)	37 (12–63)	31 (24–41)	0.76
Mechanical ventilation	46 (96%)	27 (93%)	19 (100%)	0.24
Duration of mechanical ventilation, d	22 (9–40)	30 (4–65)	20 (12–25)	0.50
ECMO	12 (25%)	11 (38%)	1 (5%)	0.01
SAPS II, without GCS calculation	853 (438–1735)	1197 (372–2226)	684 (505–914)	0.12
TISS 10	435 (187–683)	568 (165–1359)	359 (241–490)	0.13
Expenditure points for complex intensive care treatment, SAPS II+TISS 10	1329 (683–2413)	1652 (500–3482)	1074 (740–1415)	0.13
Continuous kidney replacement therapy, d	…	12 (6–36)	…	…
Cardiovascular risk factors				
Arterial hypertension	11 (23%)	7 (24%)	4 (21%)	0.80
Diabetes	1 (2%)	0 (0%)	1 (5%)	0.21
Hyperlipidemia	12 (25%)	10 (35%)	2 (11%)	0.06
Atrial fibrillation	3 (6%)	2 (7%)	1 (5%)	0.82
Laboratory data				
Elevated troponin T/L during ICU stay*	26 (72%)	17 (77%)	9 (64%)	0.40
Peak troponin T, ng/L*	54 (13–308)	174 (14–470)	53 (9–134)	0.24
Peak troponin I, ng/mL*	0.19 (0.03–0.72)	0.41 (0.06–1.23)	0.08 (0.03–0.62)	0.54
GFR, mL/min per 1.73 m^2^, at ICU discharge	97 (48–118)	73 (27–111)	114 (94–141)	0.002†
GFR, mL/min per 1.73 m^2^, at hospital discharge	97 (76–123)	94 (64–111)	112 (94–132)	0.03†
GFR, mL/min per 1.73 m^2^, at CMR	99 (81–118)	96 (72–117)	108 (95–121)	0.049†
Creatinine, mg/dL, at ICU discharge	0.8 (0.6–1.3)	1.0 (0.7–2.0)	0.7 (0.5–0.8)	0.003†
Creatinine, mg/dL, at hospital discharge	0.8 (0.6–1.1)	0.9 (0.7–1.2)†	0.7 (0.6–0.9)	0.049†
Creatinine, mg/dL, at CMR	0.8 (0.7–1.0)	0.9 (0.7–1.1)	0.7 (0.7–0.9)	0.06

Values are reported as mean±SD for parametric variables, median (interquartile range) for nonparametric continuous variables, and n (percent) for categorical variables. AKI indicates acute kidney injury; AKI+, with acute kidney injury; AKI−, without acute kidney injury; CMR, cardiac magnetic resonance; ECMO, extracorporeal membrane oxygenation; GCS, Glasgow Coma Scale; GFR, glomerular filtration rate; ICU, intensive care unit; SAPS II, Simplified Acute Physiology Score II; and TISS 10, Therapeutic Intervention Scoring System‐10.

* Troponin I/T was available in 36 of 48 participants (AKI: 22/29, non‐AKI: 14/19).

† Statistical significance.

### Myocardial Function

**Figure 2:**
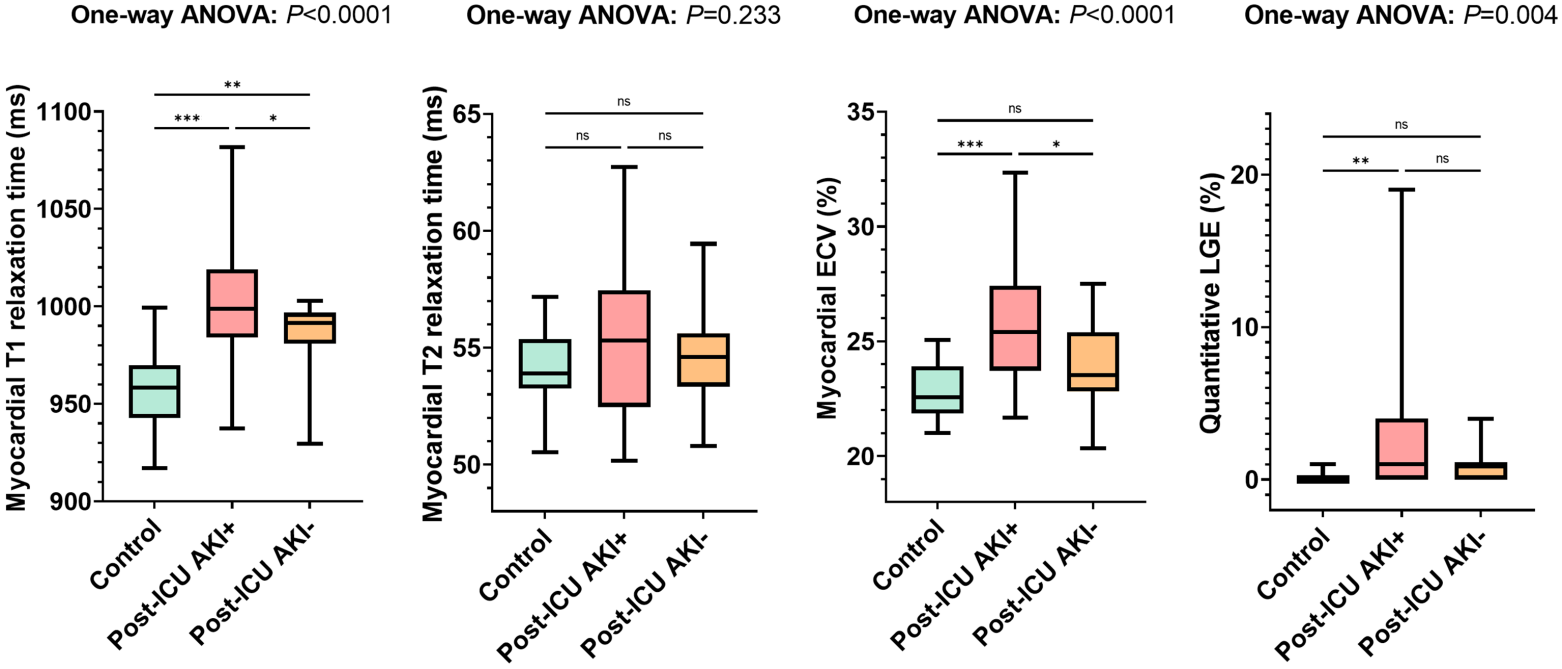
Box‐and‐whisker plots show cardiac magnetic resonance parameters for ICU survivors of critical illness with and without history of recovered acute kidney injury (AKI+ and AKI−) and healthy controls. Significance levels are indicated as follows: *P≤0.05; **P≤0.01; ***P≤0.001. ECV indicates extracellular volume fraction; ICU, intensive care unit; LGE, late gadolinium enhancement; and ns, nonsignificant.

Left ventricular ejection fraction was reduced in ICU survivors compared with healthy controls (57±6% versus 60±5%; *P*=0.03); 8 of 48 ICU survivors (17%) demonstrated a left ventricular ejection fraction of <50%. Also, ICU survivors of critical illness had impaired GLS (−20.3±3.7% versus −23.1±3.5%; *P*=0.004) and global circumferential strain (−20.3±4.4% versus −24.1±2.7%; *P*=0.001) compared with healthy controls (Table 2). Between the AKI and the non‐AKI group, there was no difference for left ventricular ejection fraction, GLS, and global circumferential strain (Table 3). Compared with healthy controls, left ventricular GLS was impaired in AKI participants (−19.9±3.8% versus −20.9±3.5%; *P*=0.008) but not in non‐AKI participants (Figure 2, Table 3). The presence of left ventricular segmental hypokinesia differed between AKI participants and healthy controls (6/29 [21%] versus 0/20 [0%]; *P*=0.03) but not between non‐AKI participants and healthy controls (1/19 [5%] versus 0/20 [%]; *P*=0.30).

**Table 2:** Clinical and Cardiac Magnetic Resonance of ICU Survivors of Critical Illness and Healthy Control Participants

Parameter	ICU survivors, n=48	Healthy controls, n=20	*P* value
General parameters			
Age, y	46±15	48±14	0.74
Sex, women	20 (42%)	9 (45%)	0.80
Body mass index, kg/m^2^	28±4	24±5	0.003
Heart rate, bpm	67±10	65±11	0.44
CMR parameters			
Pericardial effusion, ≤10 mm	10 (21%)	0 (0%)	0.03*
Pericardial effusion, >10 mm	0 (0%)	0 (0%)	…
Pleural effusion, >20 mm	2 (4%)	0 (0%)	0.35
Left ventricular ejection fraction, %	57±6	60±5	0.03*
Visual left ventricular segmental hypokinesia	7 (15%)	0 (0%)	0.07
Left ventricular end‐diastolic volume index, mL/m^2^	84±20	85±17	0.97
Cardiac index, L/min per m^2^	3.1±0.7	3.2±0.8	0.89
Left ventricular mass index, g/m^2^	49±10	46±8	0.21
Left ventricular systolic global longitudinal strain, %	−20.3±3.7	−23.1±3.5	0.004*
Left ventricular systolic global circumferential strain, %	−20.3±4.4	−24.1±2.7	0.001*
Left ventricular systolic global radial strain, %	31.6±8.9	34.0±8.8	0.31
Right ventricular ejection fraction, %	52±8	51±6	0.61
Visual myocardial edema on T2 STIR	0 (0%)	0 (0%)	…
Visual late gadolinium enhancement	10 (21%)	0 (0%)	0.03*
Ischemic pattern	5 (10%)	…	…
Nonischemic pattern	4 (8%)	…	…
Pericardial enhancement	1 (2%)	…	…
Late gadolinium enhancement, %	1 (0–3)	0 (0–0)	<0.001*
T1 relaxation time, native, ms	995±31	957±21	<0.001*
T2 relaxation time, ms	55±3	54±2	0.08
Extracellular volume fraction, %	24.9±2.5	22.8±1.2	<0.001*

Values are reported as mean±SD for parametric variables, median (interquartile range) for nonparametric continuous variables, and n (percent) for categorical variables. CMR indicates cardiac magnetic resonance; ICU, intensive care unit; and T2 STIR, T2 short‐τ inversion recovery.

* Statistical significance.

**Table 3:** Cardiac Magnetic Resonance Characteristics of ICU Survivors of Critical Illness With Recovery From AKI or Without History of AKI and Healthy Control Participants

CMR parameters	ICU survivors AKI+, n=29	ICU survivors AKI−, n=19	Healthy controls, n=20	*P* value between all groups	*P* value, AKI+ vs AKI−	*P* value, AKI+ vs controls	*P* value, AKI− vs controls
Pericardial effusion, ≤10 mm	5 (17%)	5 (26%)	0 (0%)	0.06	0.45	0.05	0.01*
Pericardial effusion, >10 mm	0 (0%)	0 (0%)	0 (0%)	…	…	…	…
Pleural effusion, >20 mm	2 (7%)	0 (0%)	0 (0%)	0.25	0.24	0.02*	…
Left ventricular ejection fraction, %	57±6	57±6	60±5	0.09	0.99	0.12	0.13
Left ventricular segmental hypokinesia, yes/no	6 (21%)	1 (5%)	0 (0%)	0.045*	0.14	0.03*	0.30
Left ventricular end‐diastolic volume index, mL/m^2^	82±22	88±17	85±17	0.55	0.51	0.88	0.83
Cardiac index, L/min per m^2^	3.1±0.7	3.3±0.7	3.2±0.8	0.64	0.62	0.87	0.91
Left ventricular mass index, g/m^2^	47±11	52±8	46±8	0.14	0.26	0.85	0.14
Left ventricular global longitudinal strain, %	−19.9±3.8	−20.9±3.5	−23.1±3.5	0.01*	0.58	0.008*	0.15
Left ventricular global circumferential strain, %	−19.8±4.3	−21.1±4.5	−24.1±2.7	0.002*	0.55	0.001*	0.049*
Left ventricular global radial strain, %	31.0±9.4	32.5±8.3	34.0±8.8	0.51	0.84	0.48	0.85
Right ventricular ejection fraction, %	53±8	50±8	51±6	0.26	0.26	0.51	0.89
Visual myocardial edema on T2 STIR	0 (0%)	0 (0%)	0 (%)	…	…	…	…
Visual late gadolinium enhancement	8 (28%)	2 (11%)	0 (0%)	0.02*	0.16	0.01*	0.14
Ischemic pattern	4 (14%)	1 (5%)	…	…	0.34	…	…
Nonischemic pattern	2 (7%)	1 (5%)	…	…	0.82	…	…
Late gadolinium enhancement, %	1 (0–4)	1 (0–1)	0 (0–0)	0.001*	0.23	<0.001*	0.02*
T1 relaxation time, native, ms	1002±33	983±21	957±21	<0.001*	0.046*	<0.001*	0.01*
T2 relaxation time, ms	55±3	55±2	54±2	0.23	0.59	0.22	0.81
Extracellular volume fraction, %	25.6±2.6	23.9±1.9	22.8±1.2	<0.001*	0.02*	<0.001*	0.25

Values are reported as mean±SD for parametric variables, median (interquartile range) for nonparametric continuous variables, and n (percent) for categorical variables. For all parameters with *P*<0.05 on intergroup analysis, additional *P* values of pairwise comparison are given. AKI indicates acute kidney injury; AKI+, with acute kidney injury; AKI−, without acute kidney injury; ICU, intensive care unit; and T2 STIR, T2 short‐τ inversion recovery.

* Statistical significance.

### Myocardial Edema and Inflammation

Neither the post‐ICU group nor the control group had focal myocardial edema, and there were no group differences in global myocardial T2 relaxation times (55±3 ms versus 54±2 ms; *P*=0.08). A small pericardial effusion (≤10 mm) was detected in 10 of 48 ICU survivors (21%) but in none of the healthy control participants (0/20, 0%; *P*=0.03).

### Myocardial Fibrosis

**Figure 3:**
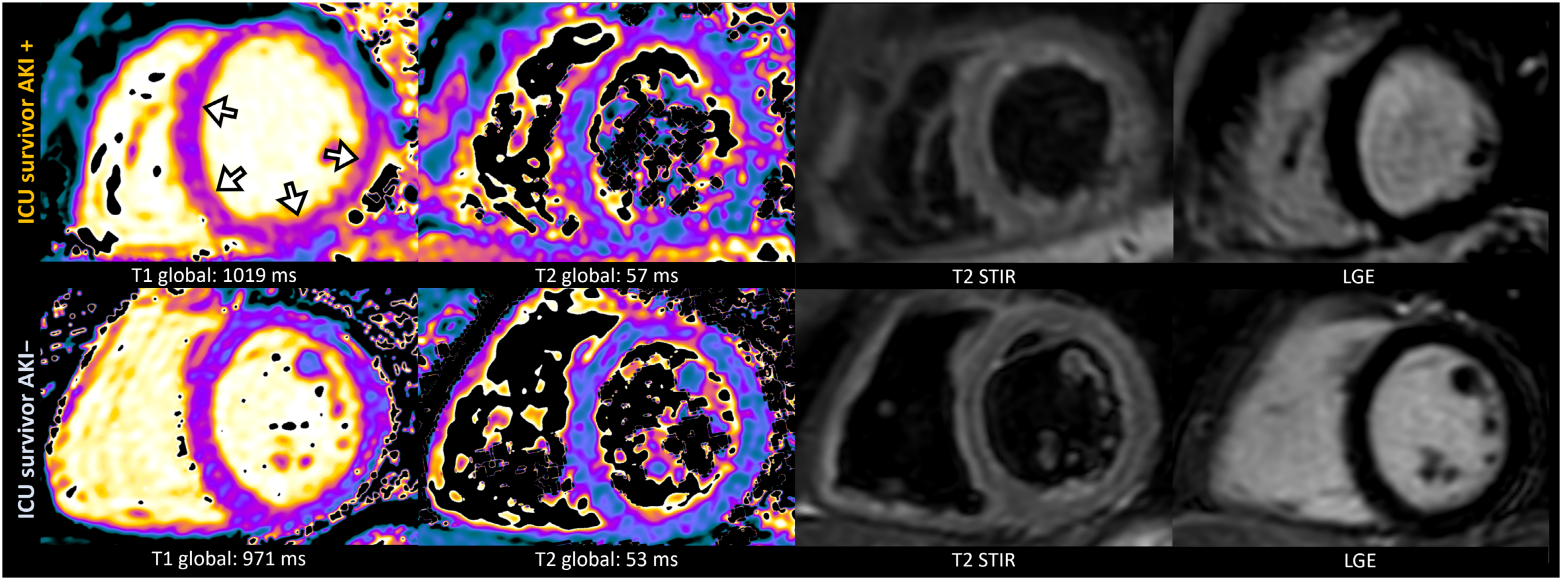
Representative images show native T1 and T2 maps for 2 ICU survivors of critical illness: a 25‐year‐old female participant with recovery of AKI displayed elevated global T1 and T2 relaxation time compared with a 33‐year‐old female participant without history of AKI (arrows show regions with elevated T1 relaxation times). No focal findings were evident on LGE and T2 STIR images in both participants. AKI indicates acute kidney injury; AKI+, with acute kidney injury; AKI−, without acute kidney injury; ICU, intensive care unit; LGE, late gadolinium enhancement; and T2 STIR, T2‐weighted short tau inversion recovery

**Figure 4:**
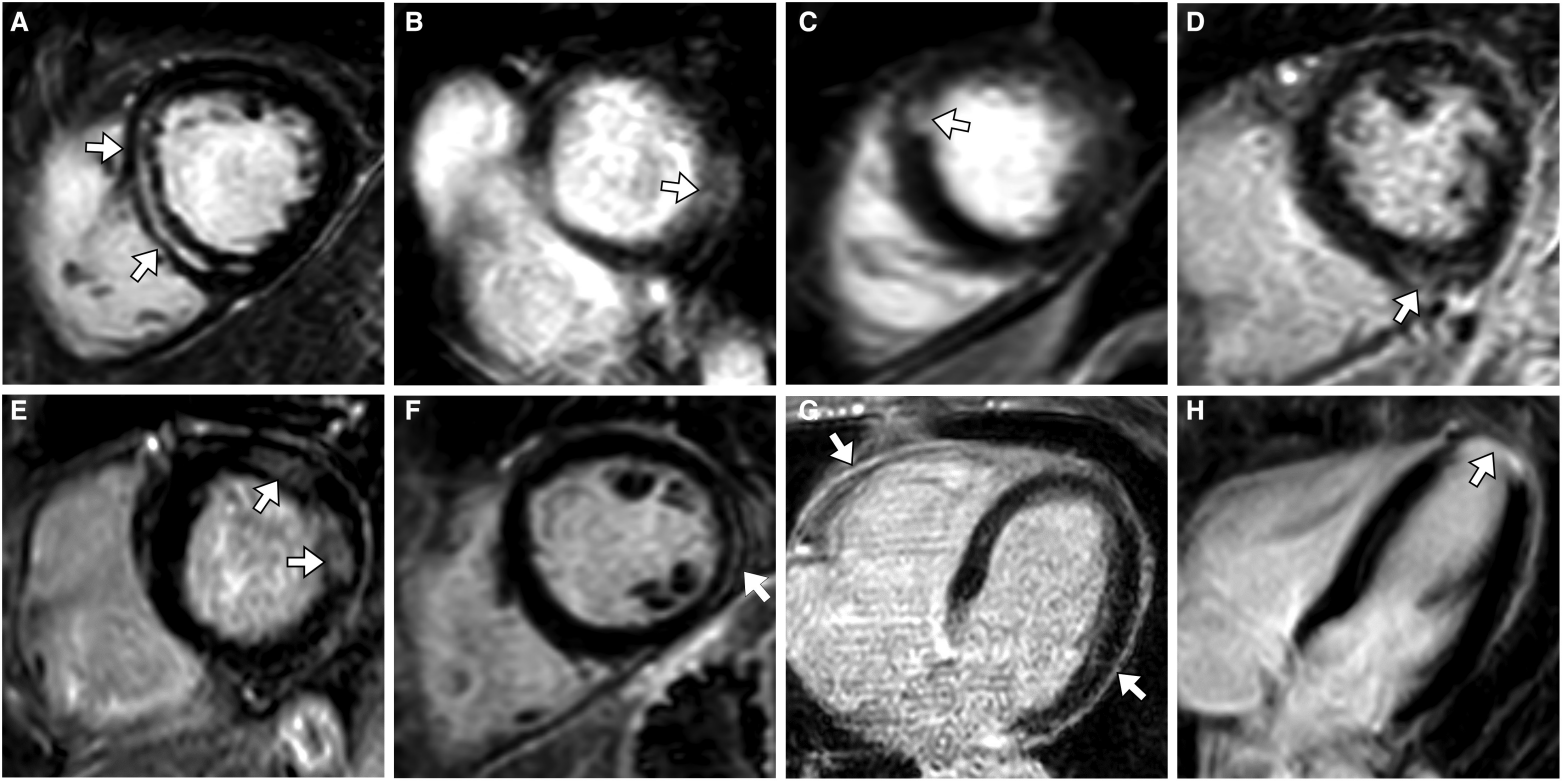
Representative examples of different enhancement patterns on late gadolinium enhancement images in intensive care unit survivors of critical illness without past medical history of cardiac disease. **A**, Extensive midwall replacement fibrosis in the basal septum. **B**, Transmural diffuse enhancement in the basal inferolateral wall (arrow). **C**, Subsegmental ischemic scar in the anteroseptal myocardium (50%–75% transmural, arrow). **D**, Subepicardial fibrosis at the inferior right ventricular insertion point (arrow). **E**, Patchy midmyocardial and subendocardial enhancement of the basal lateral wall indicating postischemic or postinflammatory fibrosis (arrows). **F**, Linear subepicardial enhancement at the midventricular inferolateral wall, indicating possible postinflammatory fibrosis (arrow). **G**, Circular pericardial thickening and enhancement indicating chronic pericarditis/fibrotic pericardial thickening (arrows). **H**, Transmural myocardial infarction of the left ventricular apex (arrow). **A** through **F**, short‐axis view. **G** through **H**, 4‐chamber view.

Myocardial T1 relaxation times (995±31 ms versus 957±21 ms; *P*<0.001) and ECV values (24.9±2.5% versus 22.8±1.2%; *P*<0.001) were significantly higher in ICU survivors compared with controls. The AKI group had higher T1 relaxation times (1002±33 ms) compared with both the non‐AKI group (983±21 ms; *P*=0.046) and the healthy control group (957±21 ms; *P*<0.001) (Figures 2 and 3). Also, AKI participants had higher mean ECV values (25.6±2.6%) compared with both the non‐AKI (23.9±1.9%; *P*=0.02) and the healthy control group (22.8±1.2%; *P*<0.001). LGE was present in 10 of 48 ICU survivors (21%) and not detectable in healthy controls (*P*=0.03). From the participants with visible LGE, 5 of 10 (50%) had an ischemic pattern, 4 of 10 (40%) had a nonischemic pattern, and 1 of 10 (10%) showed pericardial enhancement (Figure 4). Focal LGE lesions were more frequently observed in the AKI group than in the healthy control group (8/29 [28%] versus 0/20 [0%]; *P*=0.01) and the non‐AKI group (8/29 [28%] versus 2/19 [11%]; *P*=0.16). Participants with AKI had a higher LGE extent compared with healthy controls (LGE percentage, 1% [IQR, 0–4] versus 0% [IQR, 0–0]; *P*<0.001) (Figure 2).

### Correlations Between Clinical and CMR Parameters

In ICU survivors of critical illness, peak troponin T values correlated with myocardial T1 relaxation times (*r*=0.50; *P*=0.008). There were no significant correlations found between myocardial T1 relaxation times and the length of ICU stay (*r*=0.06; *P*=0.70), the duration of mechanical ventilation (*r*=0.02; *P*=0.89), or the duration of CKRT during ICU stay (*r*=0.02; *P*=0.94). Intensive care scoring Simplified Acute Physiology Score II and Therapeutic Intervention Scoring System‐10 correlated significantly with quantitative LGE values (%) (*r*=0.37; *P*=0.01). No significant differences were found between participants with and without previous extracorporeal membrane oxygenation treatment in functional (eg, left ventricular ejection fraction: 55±5% versus 57±6%, *P*=0.21; GLS: −20.7±3.5% versus −20.1±3.7%, *P*=0.61) or structural parameters (T1 relaxation times: 1005±37 ms versus 992±27 ms, *P*=0.16; T2 relaxation times: 55±3 ms versus 55±3 ms, *P*=0.71; ECV: 24.9±2.6% versus 24.9±2.5%, *P*=0.99; LGE percentage: 1% [IQR, 1–1] versus 0% [IQR, 0–0], *P*=0.07). There was no significant difference in the presence of LGE (1/8 [13%] versus 9/40 [23%], *P*=0.53) or quantitative parameters (T1 relaxation times: 993±35 ms versus 995±30 ms, *P*=0.87; T2 relaxation times: 54±2 ms versus 55±3 ms, *P*=0.20; ECV: 23.4±1.9% versus 25.2±2.5%, *P*=0.06) among ICU survivors with and without COVID‐19 during ICU treatment.

## Discussion

The main findings of this cross‐sectional study are (1) that survivors of critical illness without prior known cardiac disease had signs of myocardial fibrosis and systolic cardiac dysfunction, and (2) that these abnormalities were more pronounced after transient severe AKI. Our findings suggest that survival of acute critical illness requiring intensive care can be associated with clinically unrecognized cardiac sequelae, and those cardiac abnormalities appear to be exacerbated by severe AKI.

Critical illness causes an acute state of stress that affects the body and heart in several ways, including dysregulated proinflammatory and immunosuppressive mechanisms as well as neurohormonal and electrolyte disturbances associated with fibrotic cardiac remodeling.^3^ Regardless of its cause, focal and diffuse myocardial fibrosis can induce arrhythmia and left ventricular dysfunction affecting long‐term cardiovascular outcome.^21^, ^22^ We found positive LGE (as a marker of focal fibrosis) in 21% of ICU survivors of critical illness. Accordingly, quantitative LGE values were also elevated in the post‐ICU group. In a previous study, Ferreira et al found LGE in a nonischemic pattern to be a catecholamine‐related feature.^4^ Because upregulation of catecholamines is a common response to acute critical illness^23^ and patients in shock are often treated with vasopressors and inotropes,^24^ the nonischemic LGE patterns we observed may be fibrotic correlates after catecholamine‐induced inflammation. Critical illness has further been shown to favor atherosclerotic processes including coronary heart disease,^3^ which would be consistent with the appearance of ischemic LGE lesions in ICU survivors of critical illness. Focal ischemic LGE lesions could have also been induced by embolic myocardial infarction or vasospasm. Myocardial infarction is also a known complication of AKI.^10^

Compared with healthy controls, we found elevated myocardial T1 and ECV values in combination with normal myocardial T2 values in ICU survivors of critical illness, which may indicate the presence of diffuse myocardial fibrosis. Post‐ICU participants who recovered from AKI had the highest levels of myocardial T1 and ECV values, suggesting an association between AKI and the development of diffuse myocardial fibrosis. Profibrotic potential has been found to be associated with both chronic and acute kidney disease, with prolonged activation of the renin‐angiotensin‐aldosterone system appearing to promote cardiac fibrosis.^25^, ^26^ Moreover, in a previous CMR study, Edwards et al^27^ reported signs of diffuse myocardial fibrosis in patients with early chronic kidney disease (type 4 CRS). Findings of more pronounced myocardial fibrosis after recovered AKI are also consistent with the apparent concept that AKI is an indicator of more severe critical illness, as indicated by higher intensive care scoring for this group (eg, Simplified Acute Physiology Score II or Therapeutic Intervention Scoring System‐10).

In line with previous literature describing sepsis as a common cause of AKI,^8^ AKI frequently co‐occurred with sepsis as underlying disease in our cohort. Sepsis has also been shown to contribute to cardiac disease: In a recent CMR study, Muehlberg et al^28^ demonstrated myocardial edema and active inflammation in ICU patients during acute septic shock. None of the participants in our study showed signs of myocardial inflammation, because T2 relaxation times were within the normal reference range and no visual edema was present. The absence of active inflammation was not surprising, because only participants who recovered from critical illness were included. Our results support the hypothesis that the long‐term tissue repair processes following AKI may foster the development of myocardial fibrosis.^29^ However, because the time to CMR after ICU treatment was long in our study, ongoing chronic inflammatory processes shortly after critical illness may have been missed.^3^

Two previous echocardiography studies found left ventricular dysfunction to be common during critical illness.^30^, ^31^ Our results further suggest a long‐term impact of critical illness on cardiac function. About 17% of the ICU survivors of critical illness had a mean left ventricular ejection fraction of <50%, which, in combination with clinical symptoms, could in principle be classified as heart failure with midrange ejection fraction.^32^ Focal left ventricular hypokinesia was present in 21% of ICU survivors of critical illness after AKI, but in only 5% without AKI. Furthermore, participants with AKI had more reduced GLS and global circumferential strain values, indicating impaired myocardial contractility. Edwards et al^27^ and Hayer et al^33^ observed similar impairment of GLS in patients with early and advanced chronic kidney disease, linking kidney dysfunction to systolic impairment. Our study shows that participants with AKI have more pronounced signs of cardiac dysfunction after critical illness, even when kidney function has apparently fully recovered. Because all ICU survivors of critical illness had an unremarkable history of cardiac disease, our results demonstrate that CMR may allow for detection of previously undiagnosed but prognostically relevant myocardial findings. This observation is of particular interest, because with the COVID‐19 pandemic increased ICU admission rates were observed. Our study showed that ICU survivors of critical illness may have relevant cardiac abnormalities independent of severe COVID‐19 that may represent a possible structural component of the post–intensive care syndrome.

Some limitations have to be mentioned because of the observational form of this study. The sample size was moderate, and statistical tests may be underpowered. However, recruiting survivors of critical illness who are still eligible for CMR examination is challenging. Some differences in clinical diagnoses and treatment remained that might be confounding factors on cardiac involvement in this study. Although patients with systemic diseases with potential cardiac involvement were excluded, cardiac abnormalities might also have been present as unrecognized/subclinical conditions before the ICU stay. Also, time between ICU treatment and CMR varied. Participants in the AKI group were more frequently diagnosed with sepsis, which could be a confounding factor, because sepsis has been shown to cause myocardial injury.^6^, ^7^ More pronounced cardiac injury might have been present in patients with chronic‐to‐acute kidney disease. Lastly, our findings can only be generalized to patients with AKI requiring CKRT.

In conclusion, we showed that survivors of critical illness have unrecognized cardiac abnormalities suggestive of myocardial fibrosis and systolic dysfunction. These findings were observed in participants without known cardiac disease, indicating effects of critical illness and AKI on cardiovascular health. Transient AKI requiring CKRT was associated with more pronounced signs of myocardial fibrosis and systolic dysfunction. Because these cardiac findings may be associated with poor long‐term outcomes, survivors of critical illness could benefit from close cardiovascular monitoring. Unrecognized fibrotic and functional myocardial abnormalities in ICU survivors of critical illness may also be associated with physical impairment in postintensive care syndrome. In the present era, it is important to highlight that such cardiac abnormalities may generally occur in ICU survivors, regardless of COVID‐19 disease. Future studies should further investigate the prognostic value of such cardiac abnormalities detectable by CMR in patients after critical illness as well as in patients after AKI.

## Sources of Funding

A.I. was funded by the BONFOR Research Commission of the Medical Faculty Bonn (BONFOR‐Forschungskommission der Medizinischen Fakultät Bonn) and by the German Research Foundation (Deutsche Forschungsgemeinschaft, DFG) under Germany's Excellence Strategy EXC2151–390873048. The funders had no influence on study conceptualization and design, collection and analysis of the data, article preparation, as well as the decision to publish.

## Disclosures

The authors declare relationships with the following companies: C.C.P., speakers bureau: Guerbet, Philips Healthcare and Bayer Vital; grant support: Guerbet, Medserena AG. U.A., speakers bureau: Siemens Healthcare. J.‐C.S., grant support: Maquet Getinge. J.A.L., speakers bureau: Philips Healthcare; research consultant: Bayer AG. The authors declare that this research article was conducted in the absence of any commercial or financial relationships that could be construed as a potential conflict of interest.
